# *Cladophialophora bantiana* brain abscess and concurrent pulmonary *cryptococcus neoformans* infection in a patient twenty years after renal transplantation

**DOI:** 10.1016/j.idcr.2022.e01639

**Published:** 2022-11-05

**Authors:** Daniel Tsang, Sara Haddad, Ziver Sahin, Chairut Vareechon, Mitchell Sternlieb, Tricia Royer

**Affiliations:** a Thomas Jefferson University Hospital, USA; b Main Line Health Systems, USA

**Keywords:** Cladophialophora bantiana, Cryptococcus, Transplant, Immunocompromise, Brain abscess

## Abstract

Recipients of solid organ transplants are at risk for a variety of infections due to their immunocompromised status. The types of infections are often correlated to the timing from their transplant. After about six to twelve months, transplant recipients remain at risk for typical community acquired pathogens, late viral infections, and fungal infections including atypical molds such as *Cladophialophora bantiana. C. bantiana* is a dematiaceous fungus that has a predilection for infecting the brain and is the most common cause of cerebral phaeohyphomycosis - a term used to describe infections caused by molds that produce dark cell walls. Patients with cerebral abscesses due to *C. bantiana* infections have an estimated mortality of about 70%. Improved outcomes have been seen in patients who receive both surgical and antifungal therapy. While there are no clear guidelines on antifungal therapy, most cases have been treated with combination amphotericin B, a triazole (itraconazole, voriconazole, or posaconazole) with flucytosine sometimes in conjunction as well. This case describes a patient with *C. bantiana* brain abscess and concurrent *Cryptococcus neoformans* pulmonary infection that occurred twenty years after his kidney transplantation. He was treated successfully with two craniotomies for cerebral abscess debridement and liposomal amphotericin B followed by planned lifelong voriconazole.

## Introduction

Since the first successful organ transplant surgery in 1954, infections have been a major cause of morbidity and mortality due to the immunosuppressants needed to prevent transplant rejection [1]. The types of infections can be diverse and differ depending on the degree of immunosuppression. The immunosuppressive state varies based on the timing from transplant with the most immunosuppression usually occurring around one to six months from transplant. After six to twelve months, patients generally receive reduced or stable amounts of immunosuppression and therefore are at reduced risk for opportunistic infections. However, these patients still remain at risk for community acquired pathogens, late viral infections, and fungi including atypical molds [2].

Phaeohyphomycosis refers to a variety of infections caused by dematiaceous fungi. These fungi have dihydronaphthalene melamine in their cell walls which often lead to a brownish-yellow color visible on hematoxylin and eosin (H&E) stain. Grossly, the colonies can appear olive or black. There are numerous genera and species of fungi that cause phaeohyphomycosis that are found throughout the world in soil and decaying vegetation, particularly in warmer climates [3].

*Cladophialophora bantiana* is a dematiaceous fungus that is the most common cause of cerebral phaeohyphomycosis. However, fungal infections in the central nervous system (CNS) are uncommon, especially from fungi other than cryptococcus. Exposure may occur through inhalation of soil or decaying vegetation which can lead to pulmonary infection. Extension into the brain may occur from the sinuses or from hematogenous spread. Traumatic inoculation can also cause cutaneous disease [4]. Interestingly, *C. bantiana* infections have been found to occur at similar rates in immunocompetent hosts compared to immunocompromised hosts [5].

Due to its overall rarity in causing human disease, there are no standard guidelines on how to treat infection from *C. bantiana* in the CNS [6]. Treatment typically consists of surgical debridement, if possible, in addition to combination antifungal therapy. Antifungal regimens have consisted of liposomal amphotericin B with a triazole (usually voriconazole or itraconazole), and sometimes flucytosine as well. Despite treatment, mortality rates have been estimated to be around 70% [7].

This case describes a kidney transplant patient who developed *C. bantiana* brain abscesses despite his transplant surgery being twenty years prior. Although the mortality rates are high, this patient was successfully discharged home after two surgical debridements and antifungal therapy consisting of liposomal amphotericin B and flucytosine, followed by planned lifelong voriconazole suppression.

## Case

A 58 year old man presented to the hospital with forehead discomfort and vertigo. His past medical history was notable for non-insulin dependent diabetes mellitus, hypertension, herpes zoster infection complicated by post herpetic neuralgia and end stage kidney disease for which he underwent deceased donor kidney transplant in 2001. Prior to admission, the patient was maintained on stable doses of mycophenolate mofetil 500 mg twice daily, tacrolimus 1.5 mg twice daily, and prednisone 10 mg once daily without any recent episodes of rejection or need for steroid pulses.

**Fig. 1:**
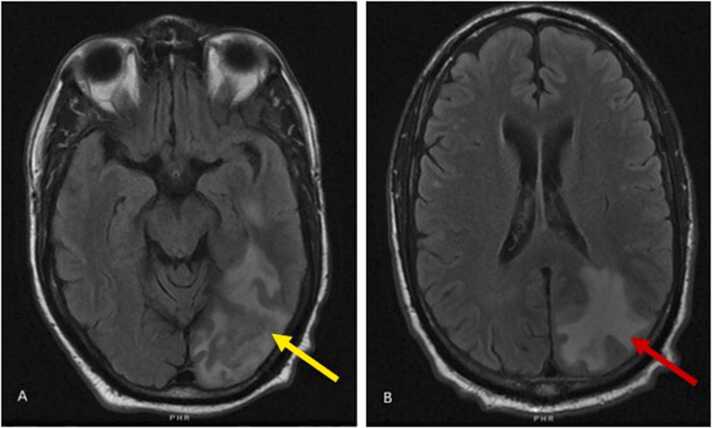
MRI brain T2 flair images – inferolateral left temporal lobe lesion with surrounding vasogenic edema (yellow arrow) and 2.7 cm occipital lesion (red arrow).

**Fig. 2:**
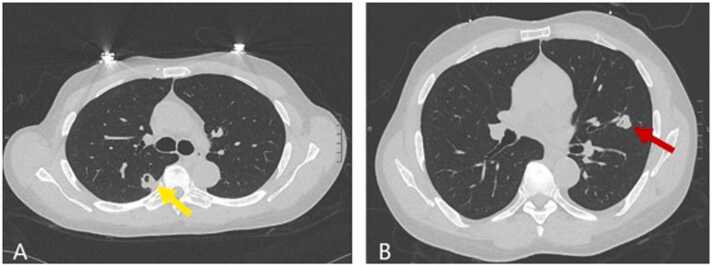
CT scan of the chest without IV contrast. A: Cavitary pulmonary mass in the superior segment of the right lower lobe measuring approximately 15 × 18 mm (yellow arrow). B: cavitary mass in the posterior left upper lobe measuring 15 × 12 mm (red arrow).

On admission, his vitals were within normal limits**.** Physical exam including his neurological assessment revealed no significant abnormalities. His bloodwork was notable for a white blood count within normal limits and a creatinine of 2.7 mg/dL, which was around his baseline (Table 1). Human Immunodeficiency Virus (HIV) screen was negative. Computed tomography (CT) of his head without intravenous contrast showed an ill-defined mass in the left occipital lobe with surrounding vasogenic edema which was concerning for abscess or metastatic disease. In addition, there was a 12 × 12 mm mass in the left temporal lobe with no midline shift noted. Magnetic resonance imaging (MRI) of the brain showed a 2.3 cm mass in the left occipital lobe (Fig. 1)**.** The findings were initially suspected to be neoplastic in nature and the patient was worked up for metastatic lesions. He underwent a CT chest that showed cavitary masses in the right lower and left upper lobes (Fig. 2). He underwent biopsy of his left upper lung lobe mass with interventional radiology which showed chronic inflammation and necrosis without evidence of malignancy. Tissue cultures grew *Cryptococcus neoformans*. His serum cryptococcal antigen titer was mildly elevated at 1:10. He was then started on flucytosine and liposomal amphotericin B 3 mg/kg daily.

**Table 1:** Laboratory Workup.

**Fig. 3:**
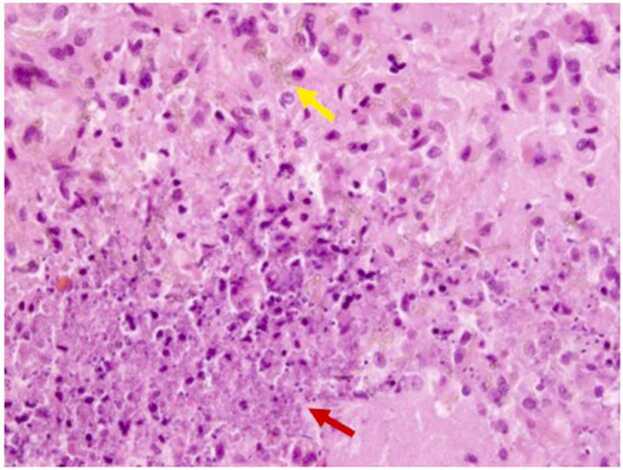
H&E stain of left necrotic brain tissue at 400X magnification. Upper half of the image shows multinucleated giant cells, golden-brown pigmented, and broad-based hyphae (yellow arrow). The lower half of the image shows blue-purple dusty nuclear debris and pink necrotic areas (red arrow). (For interpretation of the references to colour in this figure legend, the reader is referred to the web version of this article.)

**Fig. 4:**
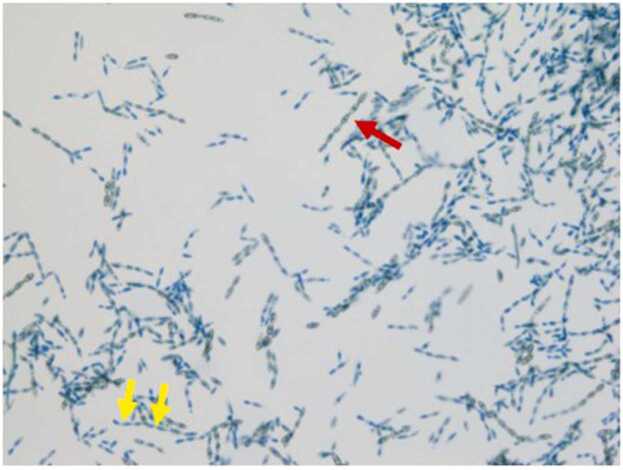
High power (40x) magnification of lactophenol cotton blue stain which show pigmented, septate hyphae (yellow arrows) with long chains of pale brown oval conidia (red arrow) with occasional branching, typical of *Cladophialophora bantiana*. (For interpretation of the references to colour in this figure legend, the reader is referred to the web version of this article.)

Five days after the initiation of therapy, he had a repeat MRI brain which showed an increase in size of his left occipital mass from 2.3 cm to 2.7 cm. Therefore, he underwent left craniotomy with resection of the brain abscess. Gross examination of the surgically resected left temporal lobe specimen showed 1.4×1.0×0.4 cm gray, pink, glistening soft tissue. H&E stained sections revealed brain tissue with areas of pink necrotic tissue surrounded by blue-purple nuclear debris, aggregates of epithelioid histiocytes, multi-nucleated giant cells, acute inflammatory infiltrate, and septate, broad-based hyphae with brown pigment (Fig. 3). These findings were consistent with necrotizing granulomatous infection indicating cerebral phaeohyphomycosis. The left temporal brain tissue was cultured onto Sabouraud Dextrose Agar, brain heart infusion agar with 5% sheep red blood cells, and Mycosel agar. After 4 days at 30°C, a velvety black mold was isolated. Microscopic examination of lactophenol cotton blue stains revealed pigmented, septate hyphae with long chains of pale brown oval conidia with occasional branching, indicating *Cladophialophora bantiana* (Fig. 4). Voriconazole was added to his regimen. Blood cultures throughout his hospitalization remained negative. After the patient was questioned about possible environmental exposures, he stated that he had “black mold” growing along his bedroom wall.

Post procedure, the patient underwent a repeat MRI brain that showed postsurgical changes in the left occipital lobe with pneumocephalus in addition to findings suggestive of superimposed ventriculitis and suspicion for a left temporal lobe abscess. He underwent repeat craniotomy with circumferential mobilization of the underlying temporal abscess that was noted to extend to the pia matter and was removed en bloc. Repeat pathology was notable for septate fungal hyphae in association with necrotizing granulomatous inflammation with intraoperative cultures again growing *C. bantiana*. Flucytosine was discontinued after 12 days of therapy and patient was maintained on voriconazole and liposomal amphotericin B. The liposomal amphotericin B was discontinued after voriconazole reached therapeutic levels. Mycophenolate was discontinued after discussion with the transplant team. Susceptibilities were obtained (Table 2), but there are currently no established minimum inhibitory concentration breakpoint interpretations. He was discharged home with an indefinite course of voriconazole 200 mg twice daily and close outpatient follow up. This case report was written one year after his hospitalization. On his most recent follow up, he endorsed compliance and no side effects with the voriconazole and felt well overall.

**Table 2:** Susceptibility testing.

Drugs	Results
Amphotericin B	0.5 mcg/mL
Itraconazole	0.03 mcg/mL
Voriconazole	1 mcg/mL
Isavuconazole	0.25 mcg/mL

## Discussion

Rates of *Cladophialophora bantiana* infection show a slight predominance for immunocompetent (59.2%) hosts compared to immunosuppressed (40.8%) hosts [5]. A recent literature review of cerebral phaeohyphomycosis due to *C. bantiana* identified eight cases in solid organ transplant patients. In those eight patients, infections ranged from 9 to 120 months from transplant surgery with the majority of infections occurring by 20 months [7]. A unique aspect of our case of *C. bantiana* brain abscess is that it occurred 20 years after his kidney transplant. In addition, he did not have any prior episodes of rejection or increased dosages in his immunosuppressants. During his hospitalization, he was continued on his dose of tacrolimus 1.5 mg twice daily and prednisone 10 mg once daily, but had his mycophenolate mofetil 500 mg twice daily discontinued.

Oftentimes, the source of exposure is unclear. There have been case reports of *C. bantiana* infected patients who were found to have the fungus growing in their walls or soil of their residence [8]. In our case, the patient reported black mold in his bedroom wall which may have been the source of his *C. bantiana* infection. Unfortunately, the mold was unable to be cultured and therefore could not be identified as the source of his infection.

In addition to this patient’s *C. bantiana* brain abscess, he also had a concurrent, albeit asymptomatic, pulmonary *Cryptococcus neoformans* infection. His first positive culture was from the biopsy of his pulmonary nodule. After it resulted, his cerebral lesion was presumed to also be *C. neoformans* abscess, even though his serum cryptococcal antigen titer was only mildly elevated to 1:10. Therefore, he was started on liposomal amphotericin B and flucytosine for presumed pulmonary *C. neoformans* with CNS dissemination. His positive cerebral *C. bantiana* culture was unanticipated by his treatment team. Due to the rarity and lack of specific symptoms of *C. bantiana* infections, diagnosis is often made unexpectedly during surgery or in many cases, post-mortem [5]. Fortunately, the liposomal amphotericin B and flucytosine that he was started on for his *C. neoformans* infection, also covered his *C. bantiana* treatment.

While the treatment for mild to moderate cryptococcal pneumonia (without meningoencephalitis) is fluconazole 400 mg once daily for 6–12 months, this would not have been appropriate for our patient. Generally, *C. bantiana* has been found to have high minimum inhibitory concentrations (MIC) to fluconazole [5], [6], [8], [9], [10], therefore fluconazole is not recommended. While the optimal antifungal therapy is unclear, some physicians have used amphotericin B, flucytosine, and either voriconazole, itraconazole, or posaconazole with some success [11]. In our case, the patient’s pulmonary cryptococcal pneumonia and cerebral *C. bantiana* abscess were both susceptible to voriconazole. Most successfully treated patients have had surgical resection of the abscess in addition to antifungal therapy [8], [12], [13].

The exact duration of antifungal therapy is unknown, but most have used at least six months of azole therapy and there have been reports of therapy for two years [4]. Our patient had a new abscess and ventriculitis several days after his first resection and ultimately needed a second resection to completely clear based on repeat imaging. He was discharged with plan to be on azole therapy lifelong, but the need will continually be reassessed with his outpatient infectious disease provider.

## Conclusion

Transplant recipients are at higher risk for infections from atypical organisms such as *Cladophialophora bantiana* and *Cryptococcus neoformans*, even decades after their transplant surgery. Diagnosis of one type of infection does not exclude concurrent infections and a high index of suspicion should remain for co-infections in immunocompromised patients. *C. bantiana* brain abscesses have a high mortality rate and successful treatment consists of both surgical resection and antifungal therapy.

## CRediT authorship contribution statement

**Daniel Tsang:** Writing – original draft preparation, **Sara Haddad:** Writing – original draft preparation, **Ziver Sahin:** Writing – original draft preparation, **Chairut Vareechon:** Writing – original draft preparation, **Mitchell Sternlieb:** Writing – reviewing and editing, **Tricia Royer:** Writing – reviewing and editing.
